# Culture supernatants from human-derived commensal bacteria alleviate DNCB-induced atopic dermatitis through modulation of inflammatory and barrier-associated pathways

**DOI:** 10.3389/fmicb.2026.1813592

**Published:** 2026-05-01

**Authors:** Jaeeun Sin, Dobin Choi, Inseong Hwang, Seongjae Kim, Heeyoun Bunch, Hangeun Kim, Dae-Kyun Chung

**Affiliations:** 1 Graduate School of Biotechnology, Kyung Hee University, Yongin, Republic of Korea; 2 Research and Development Center, Skin Biotechnology Center Co., Ltd., Yongin, Republic of Korea; 3 Department of Applied Biosciences, Kyungpook National University, Daegu, Republic of Korea

**Keywords:** anti-inflammation, atopy dermatitis, *Brachybacterium paraconglomeratum*, *Brevibacterium casei*, skin microbiome, skin-neuroimmune association

## Abstract

**Introduction:**

Atopic dermatitis (AD) is a chronic inflammatory skin disorder characterized by immune dysregulation, impaired epidermal barrier function, and recurrent episodes of itching and inflammation. Emerging evidence suggests that skin-resident microbiota influence host immune responses and may modulate AD pathogenesis. Here, we investigated the anti-inflammatory, barrier-restoring, and neuro-supportive effects of culture supernatants (CSs) derived from skin-resident bacteria.

**Methods:**

Human keratinocytes (HaCaT) stimulated with tumor necrosis factor-α (TNF-α) and interferon-γ (IFN-γ) were treated with CSs from various isolates. For in vivo evaluation, a 2,4-dinitrochlorobenzene (DNCB)-induced AD-like mouse model was utilized, receiving topical applications of the CSs. Furthermore, differentiated SH-SY5Y neuronal cells were treated with keratinocyte- or fibroblast-conditioned media, prepared after stimulation with bacterial CSs, to evaluate their neurotrophic potential.

**Results:**

CSs from Brachybacterium paraconglomeratum and Brevibacterium casei significantly suppressed interleukin-6 (IL-6) and C-C motif chemokine ligand 17 (CCL17) while restoring filaggrin expression. In keratinocytes and human dermal fibroblasts, these CSs increased brain-derived neurotrophic factor (BDNF) expression. In the DNCB-induced AD-like mouse model, topical application of B. paraconglomeratum and B. casei CSs reduced epidermal hyperplasia and immune cell infiltration, downregulated tyrosine hydroxylase (TH), and restored cutaneous BDNF, glial cell line-derived neurotrophic factor (GDNF), and filaggrin (FLG) expression. In differentiated SH-SY5Y neuronal cells, the conditioned media treatments markedly upregulated BDNF, GDNF, and nerve growth factor (NGF). Mechanistically, CS treatment inhibited p38 MAPK and JAK–STAT signaling.

**Discussion:**

Collectively, these findings demonstrate that specific skin-derived bacterial metabolites exert coordinated anti-inflammatory, barrier-reinforcing, and neurotrophic activities, thereby promoting associated changes in neurotrophic markers. Such microbial products may serve as promising biologic candidates for managing atopic dermatitis.

## Introduction

The skin microbiome consists of a diverse community of microorganisms and their genetic material that coexist on the skin surface, forming a crucial interface between the human body and its external environment. Unlike the gut microbiome, which can be effectively represented by fecal samples, the composition of the skin microbiome varies significantly across different anatomical sites, making comprehensive sampling and characterization challenging. Additionally, the relatively low microbial biomass on the skin surface complicates DNA collection for sequencing. Recent advances in metagenomic and culture-independent techniques have enabled in-depth analysis of skin microbial communities, even from limited sample quantities, thereby accelerating research in this field (De Pessemier et al., 2021).

In the skin microbiome–based healthcare industry, most approaches focus on utilizing probiotic or commensal-derived strains to restore microbial balance and alleviate disease. For example, *Staphylococcus hominis* has been developed to suppress *Staphylococcus aureus* overgrowth in atopic dermatitis (MatriSys Bioscience, n.d.), while specific *Cutibacterium acnes* subtypes have been selectively applied to modulate acne-associated lipid metabolism (NakedBiome, n.d.). In addition to live bacteria, increasing attention has been directed toward postbiotic approaches, including bacterial lysates, metabolites, and secreted factors, which offer improved safety and stability profiles. More recently, extracellular vesicles (EVs) derived from skin commensal microbiota have emerged as a promising therapeutic modality, capable of delivering bioactive molecules that modulate host immune responses and barrier function (Zhou et al., 2025; Harvey-Seutcheu et al., 2024). Despite these advances, challenges remain regarding manufacturing complexity, scalability, and consistency of defined bioactive components, highlighting the need for alternative or complementary strategies that retain biological efficacy while improving translational feasibility.

Inflammation is a natural defensive response that eliminates harmful stimuli and initiates tissue repair. However, persistent or dysregulated inflammation can lead to chronic inflammatory diseases, such as rheumatoid arthritis, lupus, and inflammatory bowel disease (Hannoodee and Nasuruddin, 2024; Xiang et al., 2023). Current anti-inflammatory therapies primarily rely on steroidal and nonsteroidal drugs, which are often limited by side effects (Del Grossi Moura et al., 2018; Juthani et al., 2017). Atopic dermatitis (AD) is a common inflammatory skin disease characterized by immune imbalance, barrier dysfunction, and recurrent eczema; however, an ideal treatment remains elusive (Mesjasz et al., 2023). Maintaining a balanced skin microbiome is increasingly recognized as critical for preventing or mitigating AD, as pathogenic *S. aureus* exacerbates inflammation, whereas commensal strains can suppress immune hyperactivation (Nakatsuji and Gallo, 2019; Koh et al., 2022).

Beyond cutaneous inflammation, AD is now recognized as a systemic disorder affecting the skin–brain axis. Patients often experience anxiety, depression, and sleep disturbances, which in turn exacerbate pruritus and inflammation (Yaghmaie et al., 2013; Beattie and Lewis-Jones, 2006). Chronic pruritus disrupts circadian rhythms, leading to fatigue, impaired cognition, and neuroimmune dysregulation. Cytokines such as IL-4, IL-13, and IL-31 not only drive skin inflammation but also influence glial activation and blood–brain barrier permeability, providing a mechanistic link between peripheral inflammation and central nervous system dysfunction (Matsuda et al., 2019; Kim et al., 2022). These findings underscore the importance of addressing neurocutaneous interactions in the pathophysiology of AD.

In this study, we prepared culture supernatants (CSs) derived from skin-resident bacteria isolated from healthy individuals and evaluated their anti-inflammatory, barrier-restorative, and neuromodulatory effects. Unlike approaches relying on live bacteria or isolated vesicular fractions, CSs represent a complex yet readily scalable mixture of microbiome-derived bioactive factors, potentially capturing synergistic effects of secreted metabolites, proteins, and vesicle-associated components. We investigated their ability to suppress inflammatory cytokine expression in keratinocytes and to restore filaggrin and neurotrophic factor (BDNF, GDNF) expression in both *in vitro* and *in vivo* AD models. Ultimately, we aimed to elucidate their role in maintaining skin–neuroimmune homeostasis.

## Materials and methods

### Skin-resident microbiota: separation and isolation

Skin swabs were collected from healthy adult volunteers at the Mariedm Skin Research Center (Seoul, Korea). The study protocol was approved by the Institutional Review Board (IRB) of Mariedm Co., Ltd. (Approval No. MDSRC-2200PR-61), and written informed consent was obtained from all participants.

Collected samples were cultured on six types of agar media: Brain Heart Infusion (BHI), De Man–Rogosa–Sharpe (MRS), Luria–Bertani (LB), Deutsche Sammlung von Mikroorganismen und Zellkulturen (DSMZ), blood agar, and potato dextrose agar (PDA). The grown colonies were identified via 16S rRNA sequencing, and the isolates were preserved for subsequent analyses. Selected strains were cultured at the laboratory scale by inoculating 1% of the stock culture into 10 mL of the appropriate medium at 37 °C. Anaerobic bacteria were cultivated using an anaerobic gas pack and jar system (MGC AnaeroPouch, Japan), and anaerobic conditions were verified with an indicator (BR0055B, BNF Korea). All bacteria were grown under optimized temperature and incubation time conditions. The cultures were adjusted to a final concentration of 1 × 10^8^ CFU/mL for further experiments.

The grown colonies were identified via 16S rRNA sequencing, and the isolates were preserved for subsequent analyses. The 16S rRNA gene sequences of the strains used in this study have been deposited in GenBank under accession numbers PZ263528 (H-Ae-5) and PZ263529 (H-Ae-9).

### Cell culture

Human keratinocytes (HaCaT) and human dermal fibroblasts (HDFs) were maintained in Dulbecco’s Modified Eagle Medium (DMEM; Gibco, USA) supplemented with 10% heat-inactivated fetal bovine serum (FBS; Life Technologies, USA) and 1% penicillin–streptomycin (P/S; GenDEPOT, USA). Cells were cultured at 37 °C in a humidified atmosphere containing 5% CO₂ and 95% relative humidity, dissociated using trypsin–EDTA (Welgene, Korea), and passaged every 3–4 days. Cells were seeded onto 12- or 96-well plates and incubated for 24 h until reaching approximately 90% confluency.

Human neuroblastoma SH-SY5Y cells (ATCC® CRL-2266™, Manassas, VA, USA) were cultured in DMEM/Ham’s F-12 medium (Gibco, USA) supplemented with 10% FBS and 1% P/S. Cells were maintained at 37 °C under 5% CO₂ and subcultured every 2–3 days. For neuronal differentiation, SH-SY5Y cells were seeded at a density of 2 × 10^5^ cells/mL and treated with 5 μM all-trans-retinoic acid (RA; Sigma-Aldrich, USA) in DMEM/F-12 supplemented with 1% FBS for 6 days, with medium changes every other day. Differentiation was confirmed by morphological changes, including neurite outgrowth and reduced proliferation, as well as increased expression of neuronal markers βIII-tubulin (Tuj1) and microtubule-associated protein 2 (MAP2). Differentiated cells were subsequently treated with culture supernatants (CSs), HaCaT-conditioned medium (HaCaT CM), or HDF-conditioned medium (HDF CM), as described in the following sections.

### Preparation of bacterial culture supernatants (CSs)

A total of nine skin-derived bacterial isolates were initially screened for their anti-inflammatory activity using keratinocytes stimulated with TNF-α and IFN-γ (Supplementary Figure 1). Among the screened isolates, H-Ae-5 (*B. paraconglomeratum*) and H-Ae-9 (*B. casei*) were selected for further investigation based on their superior and reproducible inhibitory effects on pro-inflammatory cytokine expression. Single colonies of *Brachybacterium paraconglomeratum* (H-Ae-5) and *Brevibacterium casei* (H-Ae-9) were inoculated into 10 mL of Luria-Bertani (LB) broth and cultured under optimal anaerobic conditions at 37 °C for 24 h. Similarly, *Cutibacterium acnes* (H-An-4) was grown in 10 mL of Brain Heart Infusion (BHI) broth under anaerobic conditions at 37 °C for 48 h. These specific culture media and incubation times were optimized based on the distinct growth kinetics and nutritional requirements of each strain. The incubation periods (24 h for *Actinobacteria* strains vs. 48 h for *C. acnes*) were selected to ensure that all bacterial cultures reached the stationary phase, a stage associated with the maximal accumulation of bioactive secondary metabolites. Two percent of each preculture was transferred into 1 L of the corresponding fresh medium (LB for *B. paraconglomeratum* and *B. casei*, BHI for *C. acnes*) for large-scale cultivation. After incubation, the cultures were centrifuged at 6,000 rpm for 10 min to remove bacterial cells. The resulting supernatants were collected and filtered through a 0.22 μm polyethersulfone membrane (Corning, USA) to ensure sterility, followed by lyophilization for 72 h. To ensure batch-to-batch consistency, all culture supernatants were prepared using standardized protocols under defined and reproducible culture conditions for each strain, and the final concentrations were normalized based on the dry weight of the lyophilized material. Each batch was reconstituted in sterile PBS to a defined concentration prior to use. Unless otherwise indicated, bacterial CSs were used at a standard working concentration of 100 μg/mL, which was selected based on preliminary experiments demonstrating consistent biological activity without detectable cytotoxicity under the tested conditions. For specific assays requiring enhanced stimulation of neurotrophic factor expression (e.g., BDNF and GDNF), a higher concentration (1 mg/mL) was applied to ensure sufficient induction of measurable transcriptional responses.

### Preparation of HaCaT- and HDF-conditioned media (CM)

HaCaT and HDF cells were seeded in 6-well plates at a density of 1 × 10^5^ cells per well and incubated in complete DMEM until reaching 80–90% confluence. The cells were then washed twice with PBS and treated with bacterial CSs at a concentration of 1 mg/mL for 24 h. After treatment, the culture media were collected and centrifuged at 3,000 rpm for 5 min to remove cellular debris. The resulting supernatants were filtered through a 0.22 μm membrane filter, yielding HaCaT-conditioned medium (HaCaT CM) and HDF-conditioned medium (HDF CM). The conditioned media were stored at −80 °C until further use in neuronal cell experiments.

### Morphological characterization by scanning electron microscopy (SEM)

Selected bacterial isolates were cultured under optimal conditions and lyophilized using a freeze-dryer. Morphological features were observed using a field-emission scanning electron microscope (FE-SEM; LEO SUPRA 55, Carl Zeiss, Germany) at Kyung Hee University, Korea.

### Enzyme-linked immunosorbent assay (ELISA)

HaCaT cells (3 × 10^4^ cells/mL) were seeded into 96-well plates and incubated for 24 h. The cells were then treated with the indicated doses of bacterial CSs for 16 h, followed by stimulation with tumor necrosis factor-α (TNF-α, 10 ng/mL) and interferon-γ (IFN-*γ*, 10 ng/mL) for 24 h. Supernatants were collected and diluted 1:5 for interleukin-6 (IL-6) analysis, while undiluted samples were used for CCL17/TARC detection. IL-6 levels were measured using the BD OptEIA Human IL-6 ELISA Kit (BD Biosciences, Cat. No. 555220), and CCL17/TARC levels were measured using the Human CCL17/TARC DuoSet ELISA Kit (R&D Systems, Cat. No. DY364), following the manufacturers’ protocols.

### Cell viability assay

HaCaT cells (3 × 10^4^ cells/mL) were seeded in 96-well plates and incubated for 24 h. The cells were then treated with bacterial CSs for 16 h, followed by TNF-α and IFN-γ (10 ng/mL each) for 24 h. Cell viability was assessed using the MTT assay (1 mg/mL; Invitrogen, USA), and absorbance was measured at 550 nm using an Epoch microplate reader (BioTek Instruments, USA).

### MAPK phosphorylation antibody array

Mitogen-Activated Protein Kinase (MAPK) pathway activation was analyzed using a Human MAPK Phosphorylation Antibody Array (Abcam, Cat. No. ab211061), which targets 17 signaling proteins, including Akt, Extracellular signal-regulated kinase (ERK)1/2, p38, c-Jun N-terminal kinase (JNK), cAMP-response element binding protein (CREB), and Signal Transducer and Activator of Transcription (STAT)-related kinases. HaCaT cell lysates were collected following CS treatment (18 h) and cytokine stimulation (24 h). The arrays were performed according to the manufacturer’s protocol, including blocking (30 min), sample incubation (16 h), detection antibody incubation (2 h), and HRP–anti-rabbit IgG incubation (2 h). Densitometric analysis was conducted using ImageJ software (NIH, USA).

### Western blot analysis

HaCaT cells (4 × 10^5^ cells/mL) were treated with 1 mg/mL of CSs from H-An-4, H-Ae-5, and H-Ae-9 for 16 h, followed by stimulation with TNF-α/IFN-γ for 0–120 min. Cells were washed twice with PBS and lysed in 2 × Laemmli buffer, then boiled for 5 min at 100 °C. Proteins were separated on 12% SDS–polyacrylamide gels and transferred to Polyvinylidene Fluoride (PVDF) membranes (Millipore, USA). Membranes were blocked with 5% skim milk in Tris Buffered Saline with Tween 20 (TBST) for 1 h at room temperature and incubated overnight at 4 °C with primary antibodies against phospho-ERK1/2 (9,101 L), phospho-p38 (9211S), phospho-CREB (9198S), and phospho-STAT1 (9167S) (Cell Signaling Technology, USA). After washing, membranes were incubated with HRP-conjugated secondary antibody (Santa Cruz, sc-2357, 1:1000) for 2 h, and protein bands were visualized using EZ-Western Lumi Pico (DoGenBio, Korea) and an iBright 750 imaging system (Thermo Fisher Scientific, USA).

### Mouse study

Animal experiments were approved by the Institutional Animal Care and Use Committee of Corestemchemon, Korea (Approval No. CHEM-2024-IA0245-00). Five-week-old male BALB/c mice (19 ± 1 g; OrientBio, Korea) were housed under specific pathogen-free conditions (23 °C, 12 h light/dark cycle) with free access to food and water. After a one-week acclimation period, the dorsal skin was shaved, and the mice were randomly divided into six groups (n = 6 per group): Control, 2,4-Dinitrochlorobenzene (DNCB), DNCB + Dexamethasone, DNCB + H-An-e, DNCB + H-Ae-5, and DNCB + H-Ae-9.

AD-like lesions were induced by applying 100 μL of 1% DNCB (Sigma-Aldrich, USA) dissolved in an acetone/olive oil (4:1) mixture for sensitization, followed by 2.5% DNCB every 3 days for 2 weeks, and then 0.2% DNCB thereafter. Starting from week 2, bacterial CSs (10 mg/kg) or dexamethasone (5 mg/kg) were topically administered daily. Body weight, food intake, and photographs were recorded on days 2, 9, 16, 23, and 30. At the endpoint, mice were anesthetized with isoflurane, and blood was collected via cardiac puncture. Serum was separated by centrifugation at 2,000 rpm for 20 min and analyzed for IgE analysis using the BD OptEIA Mouse IgE ELISA Kit (Cat. No. 555248). Mice were euthanized by CO₂ inhalation, and dorsal skin tissues were excised. Portions of the tissue were fixed in 10% formalin for histological examination, while others were homogenized in RIPA buffer containing protease and phosphatase inhibitors (Sigma-Aldrich, PPC1010) for Western blot analysis of MAPK signaling.

### Histological analysis

Fixed skin tissues were embedded in paraffin, sectioned, and stained with hematoxylin and eosin (H&E). Epidermal thickness was measured under 250 × magnification, and images were analyzed using ImageJ software.

### Immunofluorescence staining for tyrosine hydroxylase (TH) and keratin 14 (K14)

Skin tissues from DNCB-induced and control mice were fixed in 4% paraformaldehyde at 4 °C overnight, cryoprotected in sucrose solutions ranging from 10 to 30%, embedded in OCT compound (Sakura Finetek, USA), and sectioned at a thickness of 7 μm using a Leica CM1950 cryostat (Germany). Sections were permeabilized with 0.2% Triton X-100 for 10 min and blocked with 5% normal goat serum for 1 h. Slides were incubated overnight at 4 °C with anti-TH (1:200; Invitrogen, OPA1-04050) and anti-K14 (1:300; Biolegend, #906004) primary antibodies. After washing, samples were incubated for 1 h with Alexa Fluor 488–conjugated goat anti-rabbit IgG and Alexa Fluor 594–conjugated goat anti-mouse IgG secondary antibodies (1:500; Invitrogen, USA), counterstained with DAPI (1 μg/mL), mounted with Fluoromount-G (SouthernBiotech, USA), and visualized using a Zeiss LSM 800 confocal laser scanning microscope (Germany).

### Statistical analysis

All data are presented as mean ± standard deviation (SD) from at least three independent experiments. Statistical significance among groups was determined using one-way ANOVA followed by Tukey’s *post hoc* test. A *p*-value of less than 0.05 was considered statistically significant. Statistical analyses were performed using GraphPad Prism 5 (GraphPad Software, USA).

## Results

### Isolation and identification of skin microbiota

Skin swabs were obtained from healthy donors, and bacterial isolates were cultured on various media, including BHI, MRS, LB, DSMZ, blood agar, and PDA. The isolates were taxonomically identified based on 16S rRNA gene sequencing, which revealed that the predominant species were *Staphylococcus epidermidis* (60%), *Staphylococcus cohnii* (10%), *Brachybacterium paraconglomeratum* (10%), *Staphylococcus warneri* (10%), and *Micrococcus luteus* (10%). To evaluate their biological relevance, the anti-inflammatory activity of each isolate was assessed by measuring IL-6 and CCL17 production in stimulated HaCaT keratinocytes (Supplementary Figure 1). Based on these screening results, *C. acnes* (designated H-An-4), *B. paraconglomeratum* (H-Ae-5), and *B. casei* (H-Ae-9) were selected for further investigation due to their differential immunomodulatory profiles.

**Figure 1:**
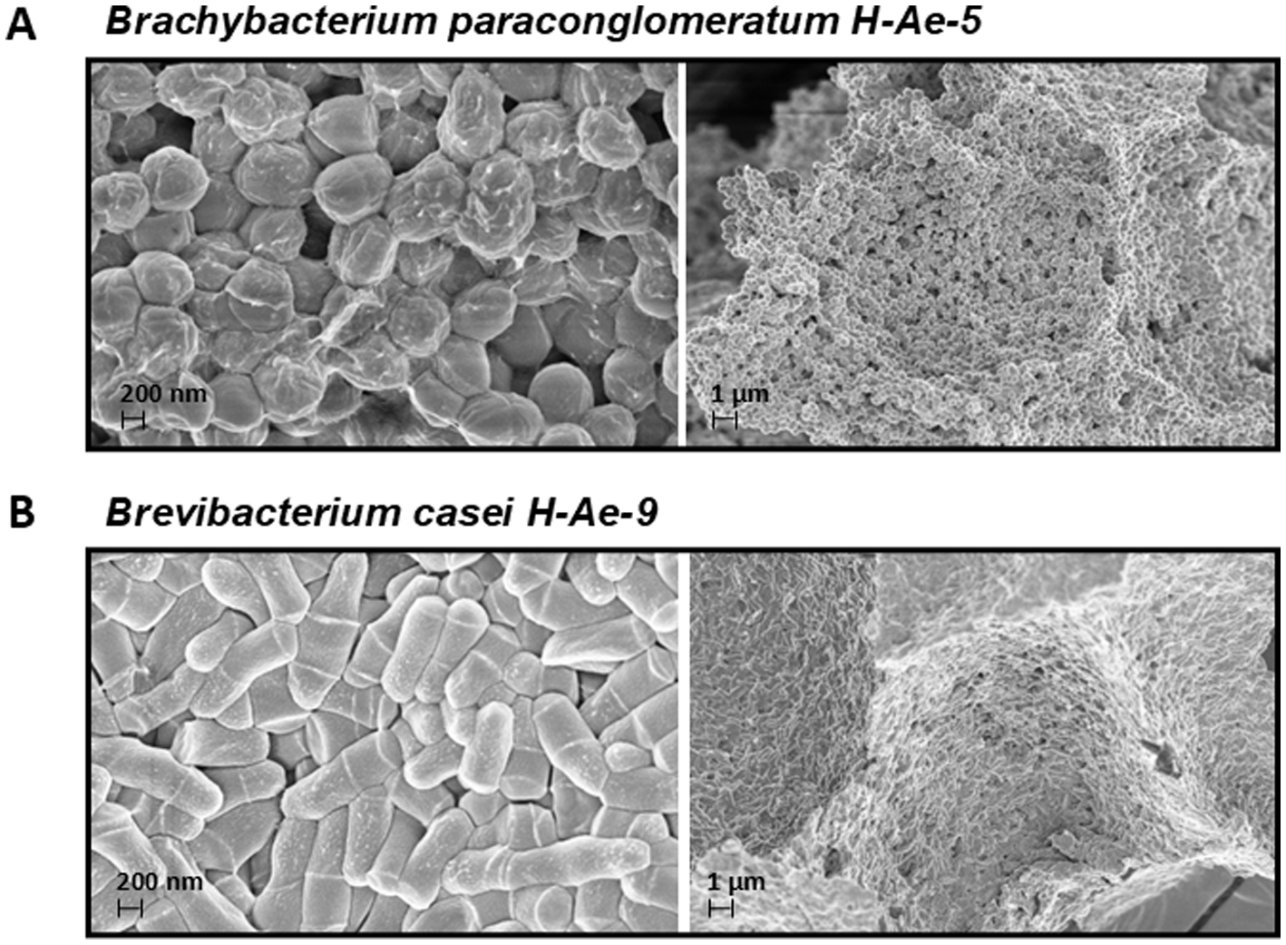
Scanning electron microscopy (SEM) images of skin-resident bacteria isolated and identified from humans. **(A)** *B. paraconglomeratum* (H-Ae-5). **(B)** *B. casei* (H-Ae-9). The images on the left are magnified at 40,000x, while those on the right are magnified at 5,000x.

Scanning electron microscope (SEM) observations were conducted on H-Ae-5 and H-Ae-9. H-Ae-5 exhibited a staphylococcal-like arrangement, forming spherical cocci aggregated by viscous extracellular material (Figure 1A). In contrast, H-Ae-9 displayed a rod-shaped morphology and did not form clusters. Both species exhibited regular shapes, with each typically displaying two or more lines (Figure 1B).

The bacterial isolates H-Ae-5 and H-Ae-9 were taxonomically classified through 16S rRNA gene sequencing, which confirmed their identity as *Brachybacterium paraconglomeratum* (H-Ae-5) and *Brevibacterium casei* (H-Ae-9), respectively. Both strains have been formally deposited in the Korean Collection for Type Cultures (KCTC) under the accession numbers KCTC 16110BP for H-Ae-5 and KCTC 16111BP for H-Ae-9, ensuring their long-term preservation and accessibility for future research.

### Anti-inflammatory effects of culture supernatants (CSs) derived from skin microbiota on HaCaT cells

**Figure 2:**
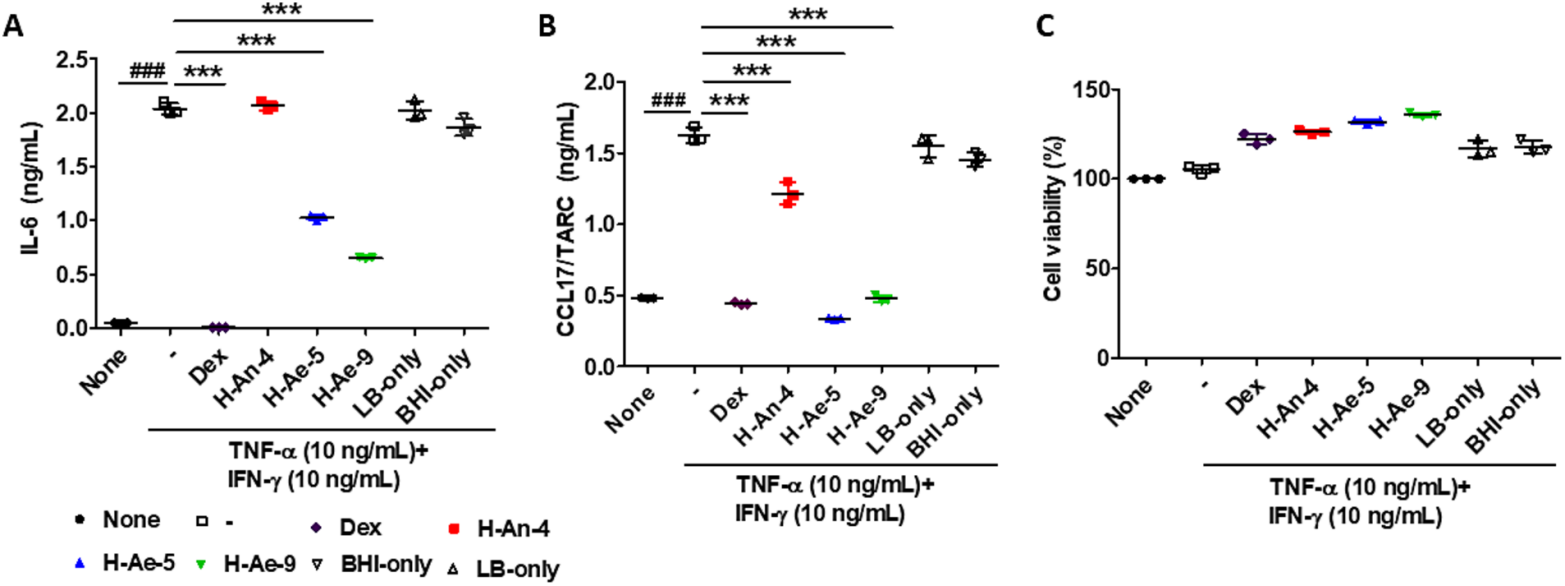
Culture supernatants (CSs) derived from skin microbiota inhibited the production of cytokines induced by TNF-α and IFN-γ (TI). HaCaT cells were pretreated with 100 μg/mL of CSs for 18 h followed by stimulation with 10 ng/mL of TNF-α and 10 ng/mL of IFN-γ for 24 h. The levels of IL-6 **(A)** and CCL17/TARC **(B)** in the culture supernatants were measured using ELISA. **(C)** The remaining cells were subjected to a cell viability assay using the WST-1 reagent. Data are presented as the mean ± standard deviation (S.D.) and were statistically analyzed using one-way ANOVA followed by Tukey’s multiple comparison test. ^###^*p* < 0.05 when comparing the TNF-α + IFN-γ-stimulated group with the treated group. Dex, dexamethasone; H-An-4, *C. acnes*; H-Ae-5, *B. paraconglomeratum*; H-Ae-9, *B. casei*.

Stimulation of HaCaT cells with TNF-α and IFN-γ (collectively referred to as TI) markedly increased the production of the pro-inflammatory cytokines IL-6 and CCL17, whereas the positive control, dexamethasone, effectively suppressed their expression. Pretreatment with culture supernatants (CSs) derived from skin microbiota significantly attenuated this inflammatory response. Specifically, CSs from H-Ae-5 and H-Ae-9 significantly suppressed TI-induced IL-6 production in HaCaT cells, to levels comparable with those observed following dexamethasone treatment (Figure 2A). In contrast, the CS from H-An-4 did not reduce TI-induced IL-6 production in HaCaT cells, indicating no significant inhibitory effect.

All three bacterial CSs significantly inhibited CCL17 secretion, with H-Ae-5 and H-Ae-9 showing slightly stronger effects than H-An-4; however, all three showed suppressive effects comparable to those of dexamethasone (Figure 2B). These results suggest that the anti-inflammatory activity of H-An-4 CS appears to be cytokine-specific, whereas the CSs from H-Ae-5 and H-Ae-9 exhibit broader inhibitory effects on inflammatory mediators. Importantly, treatment of HaCaT cells with bacterial CSs for 18 h followed by TI stimulation for 24 h did not reduce cell viability (Figure 2C), confirming that the observed decreases in IL-6 and CCL17 levels were not attributable to cytotoxicity.

Treatment with LB-only or BHI-only did not significantly alter IL-6 or CCL17 production compared to the stimulated control group. In addition, neither LB nor BHI treatment induced detectable cytotoxicity, indicating that the culture media themselves did not exert appreciable effects on inflammatory cytokine expression or cell viability.

### Skin microbiota–derived culture supernatants inhibit TI-induced activation of the p38 and STAT1 pathways

**Figure 3:**
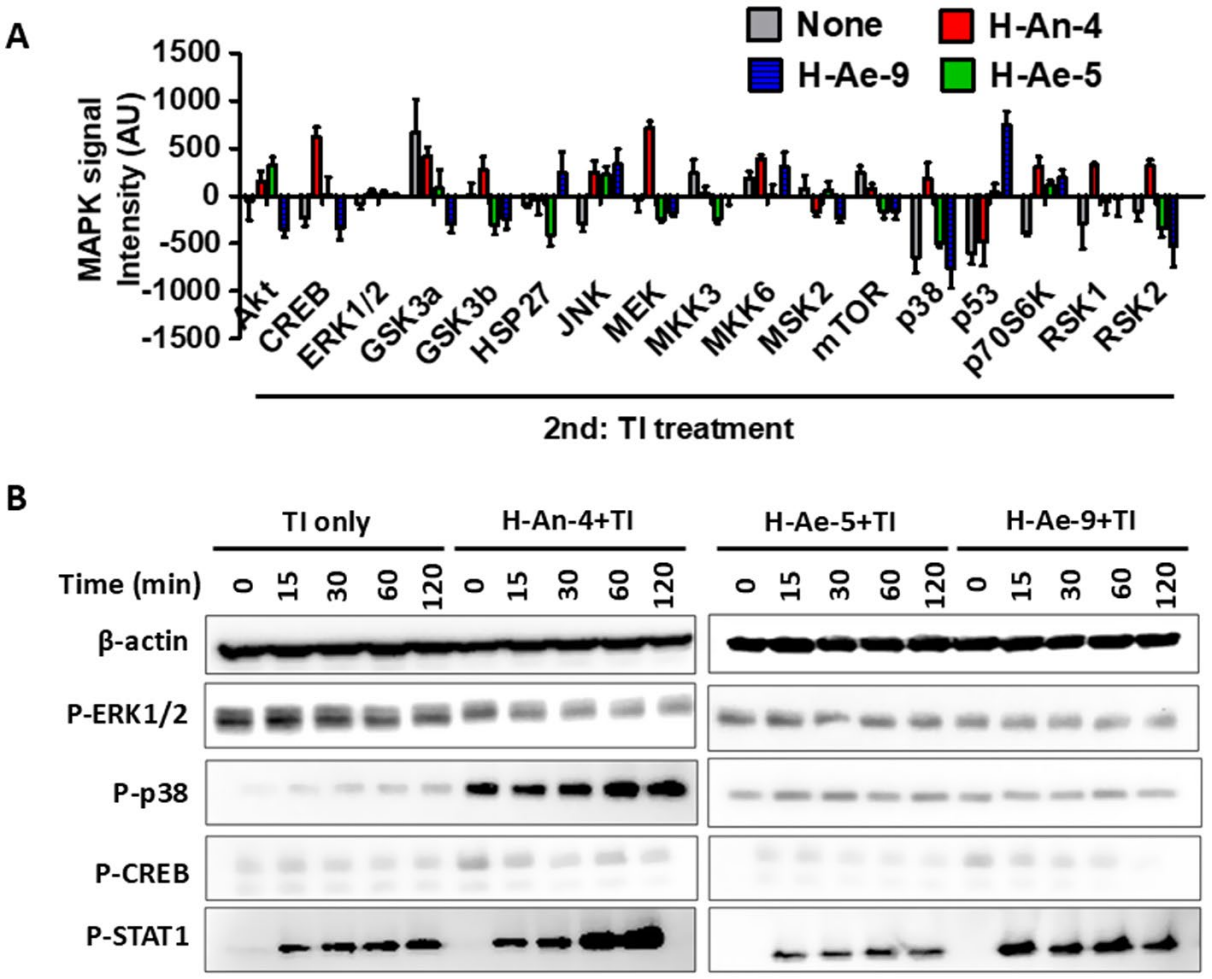
Signaling variation induced by skin microbiota. **(A)** HaCaT cells were treated with 100 μg/mL of CSs for 18 h followed by treatment with 10 ng/mL of TI for 24 h. Cell lysates were then analyzed using a MAPK array. **(B)** HaCaT cells were treated with 100 μg/mL of CSs for 18 h followed by 10 ng/mL of TI for the indicated times. WB analysis was conducted on the cell lysates.

To elucidate the signaling mechanisms underlying the anti-inflammatory effects of skin microbiota, we examined the activation profiles of multiple kinases in TI–stimulated HaCaT cells using a MAPK phosphorylation antibody array. Pretreatment with CS from H-An-4 followed by TI stimulation markedly increased the phosphorylation of CREB, GSK3β, MEK, MKK6, p38, p70S6K, RSK1, and RSK2, indicating strong activation of MAPK-associated inflammatory cascades (Figure 3A). In contrast, CSs from H-Ae-5 and H-Ae-9 significantly attenuated these phosphorylation events, suggesting suppression of IL-6 and CCL17 production through downregulation of MAPK signaling. In pathways involving HSP27, MKK3, MSK2, mTOR, and p53, H-An-4 CS showed minimal activation, whereas H-Ae-5 and/or H-Ae-9 CSs exhibited moderate stimulation in TI-treated cells. Collectively, the array analysis identified CREB, MEK, MKK6, ERK1/2, p38, and p70S6K as key nodes mediating TI-induced inflammation and its inhibition by skin microbiota–derived CSs.

To validate these array findings, Western blot analysis was conducted to assess the phosphorylation of ERK1/2, p38, CREB, and STAT1, which are key components of the IFN-*γ*–responsive signaling cascade. As shown in Figure 3B, H-An-4 CS combined with TI significantly enhanced p38 phosphorylation, whereas this activation was nearly completely suppressed by treatment with H-Ae-5 or H-Ae-9 CS. Similarly, pretreatment with H-An-4 CS increased STAT1 phosphorylation beyond the levels observed with TI alone, while H-Ae-5 CS strongly inhibited STAT1 activation. H-Ae-9 CS also reduced STAT1 phosphorylation, although to a lesser extent than H-Ae-5. These results indicate that skin microbiota–derived CSs attenuate TI-induced inflammatory signaling by selectively inhibiting the p38 MAPK and STAT1 pathways, thereby reducing the downstream production of the pro-inflammatory cytokines IL-6 and CCL17.

### Culture supernatants alleviated AD-like skin symptoms in mice

**Figure 4:**
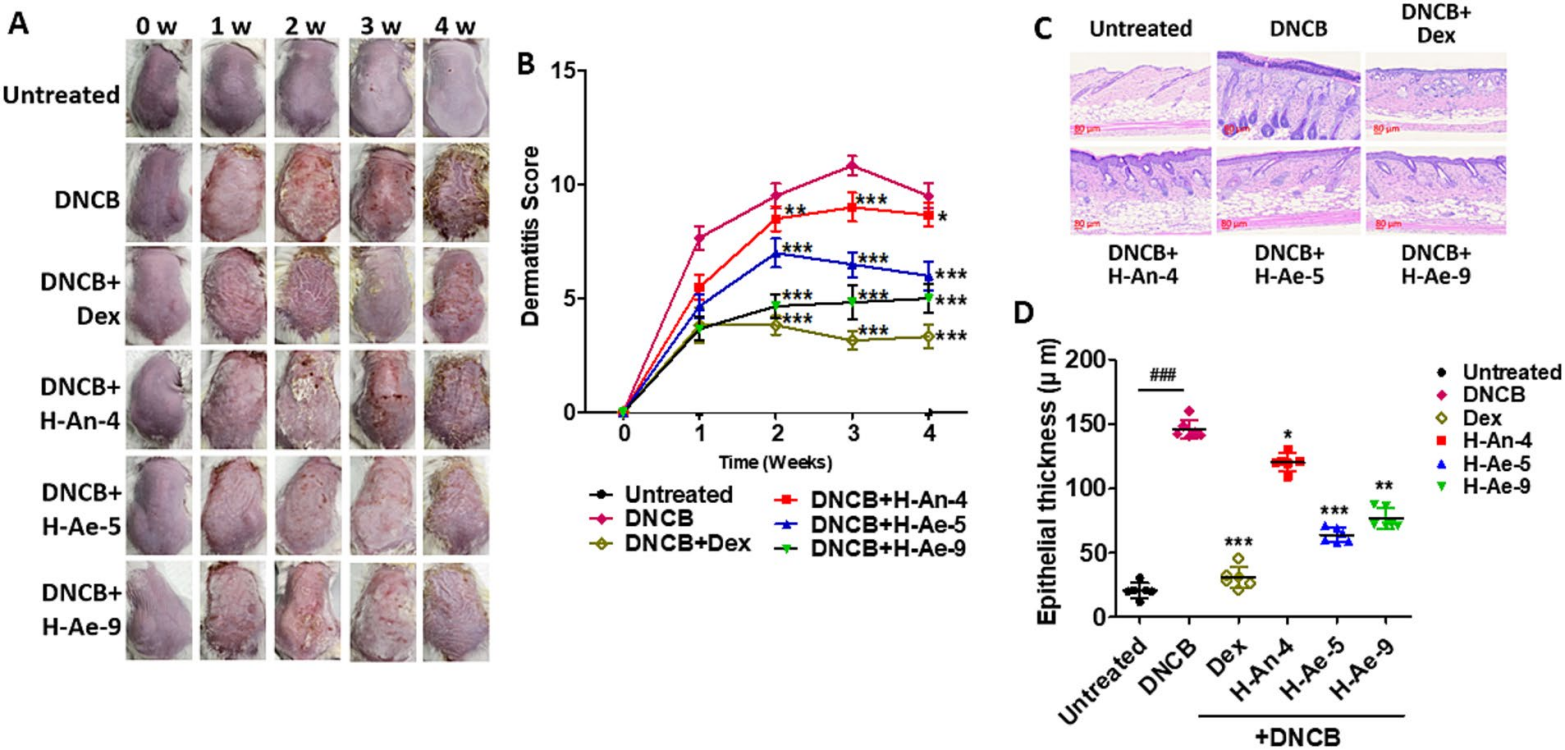
CS-mediated alleviation of AD symptoms. Mice (*n* = 6 per group) were treated with DNCB to induce atopic-like symptoms and subsequently received CSs and dexamethasone treatment for 4 weeks. **(A)** Photographs of the atopic dermatitis lesions in the mice were taken at one-week intervals. **(B)** Dermatitis scores (0–12 scale) were evaluated weekly based on erythema/hemorrhage, edema, excoriation/erosion, and scaling/dryness. **(C,D)** Epidermal thickness was analyzed following hematoxylin and eosin **(H&E)** staining **(C)** and quantified using digital image analysis software (ImageJ, NIH) **(D)**. Scale bars indicate 80 μm. *p < 0.05, **p < 0.01, ***p < 0.001, compared to the DNCB group only. ^###^*p* < 0.05 when comparing the DNCB group with the untreated group.

Topical application of CSs derived from H-Ae-5 and H-Ae-9 markedly alleviated DNCB-induced AD-like skin lesions compared to the H-An-4 CS and DNCB-only groups (Figure 4A). Mice treated with H-Ae-5 and H-Ae-9 exhibited visibly reduced erythema and excoriation, along with attenuated crust formation. In contrast, H-An-4-treated mice showed persistent erythematous and erosive lesions comparable to those observed in the DNCB-only group. Among the tested fractions, H-Ae-9 demonstrated the most pronounced improvement in gross skin appearance. Consistent with these findings, dermatitis scores were significantly reduced in the H-Ae-5 and H-Ae-9 groups throughout the experimental period (Figure 4B). Although dexamethasone treatment initially suppressed lesion severity during the first 3 weeks, prolonged administration resulted in a rebound increase in skin inflammation. Moreover, systemic adverse effects including reduced food intake and body weight loss were observed in the dexamethasone-treated mice (Supplementary Figure 2), consistent with the well-documented side effects of chronic steroid exposure.

Histopathological evaluation revealed that CSs from H-Ae-5 and H-Ae-9 effectively mitigated epidermal hyperplasia and hyperkeratosis induced by DNCB (Figure 4C). In the H-Ae-5 CS-treated group, both epidermal thickness and the stratum corneum layer were markedly reduced compared to the DNCB-only group. Although tissues treated with H-Ae-9 CS exhibited slightly thicker keratin layers than those treated with H-Ae-5, the overall tissue morphology was significantly improved relative to the untreated AD model. Conversely, treatment with H-An-4 CS resulted in a thickened epidermis with extensive cornified layers, resembling the pathological features observed in the DNCB group. Quantitative analysis confirmed that DNCB treatment significantly increased epidermal thickness across all experimental groups, consistent with hyperkeratotic remodeling (Figure 4D). In contrast, treatment with H-Ae-5 and H-Ae-9 CSs markedly reduced epidermal hypertrophy and normalized keratinocyte layering. Although dexamethasone restored near-normal architecture, it exhibited systemic toxicity. Collectively, these findings demonstrate that skin microbiota–derived CSs, particularly those from H-Ae-5 and H-Ae-9, alleviate AD-like symptoms and histopathological changes by reducing epidermal hyperplasia and promoting barrier restoration in DNCB-induced murine skin.

### Serum IgE levels decreased following treatment with CSs derived from skin microbiota

**Figure 5:**
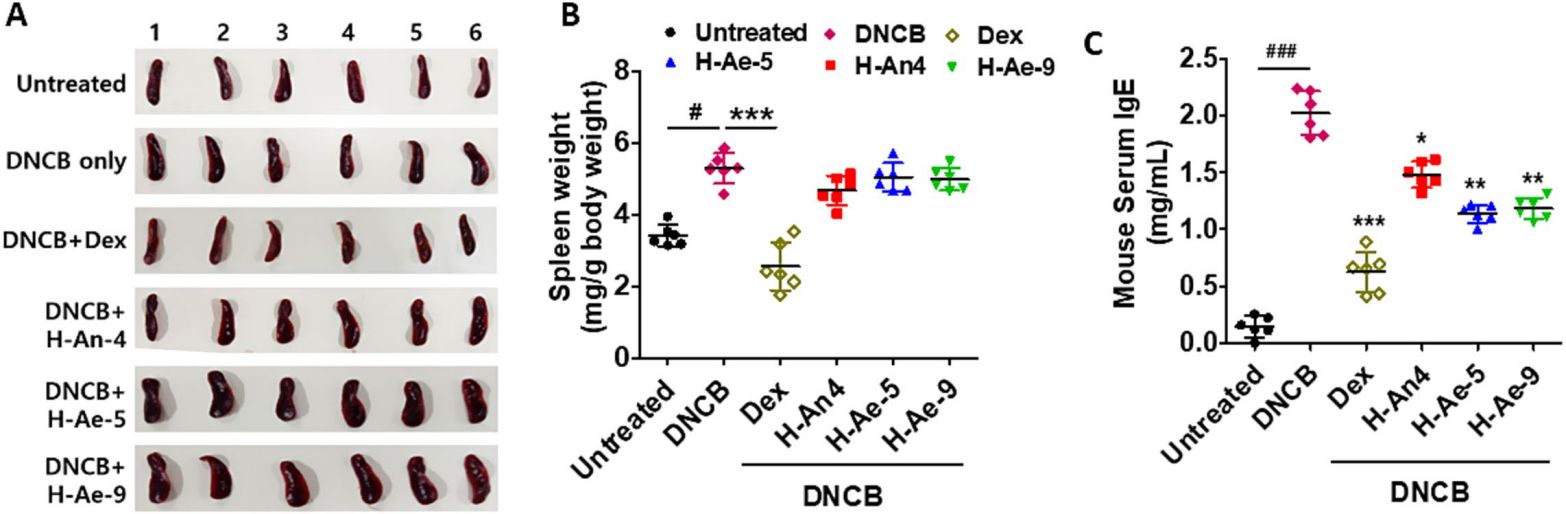
CSs reduce serum IgE levels in an atopic-like mouse model. Spleens were isolated from the mice, and their sizes are presented in images **(A)**, while the weights of the spleens were measured **(B)**. **(C)** The serum IgE levels in the mice were quantified using an ELISA kit. Data are presented as the mean ± SD and were statistically analyzed using one-way ANOVA followed by Tukey’s multiple comparison test. ^*^p < 0.05, **p < 0.01, ***p < 0.001, compared to the DNCB group only. ###*p* < 0.05 when comparing the DNCB group with the Untreated group.

To evaluate the systemic immunomodulatory effects of bacterial CSs, spleen morphology and serum IgE levels were assessed in DNCB-induced AD-like mice. As shown in Figures 5A,B, exposure to DNCB markedly increased spleen size and relative spleen weight (mg/g body weight) compared with the control group, indicating systemic immune activation. Mice treated with CSs from H-An-4, H-Ae-5, or H-Ae-9 also exhibited elevated spleen weight compared with controls, suggesting a sustained but moderated immune stimulation. No significant differences in spleen weight were observed among these three CS-treated groups. In contrast, dexamethasone-treated mice showed a pronounced reduction in spleen size and relative spleen weight, consistent with steroid-induced immunosuppression.

Serological analysis revealed that total IgE was undetectable in control mice but markedly elevated following DNCB exposure (Figure 5C). Dexamethasone administration reduced serum IgE to near-baseline levels, while treatment with CSs from H-Ae-5 and H-Ae-9 modestly lowered IgE concentrations compared to the H-An-4 CS-treated group, although the difference did not reach statistical significance. Collectively, these findings indicate that skin microbiota-derived CSs partially attenuate systemic allergic responses, as evidenced by reduced IgE production and moderated spleen enlargement in DNCB-induced AD-like mice.

### The anti-inflammatory effects of skin microbiota–derived CSs were mediated by the inactivation of STAT1 and p38 signaling pathways

**Figure 6:**
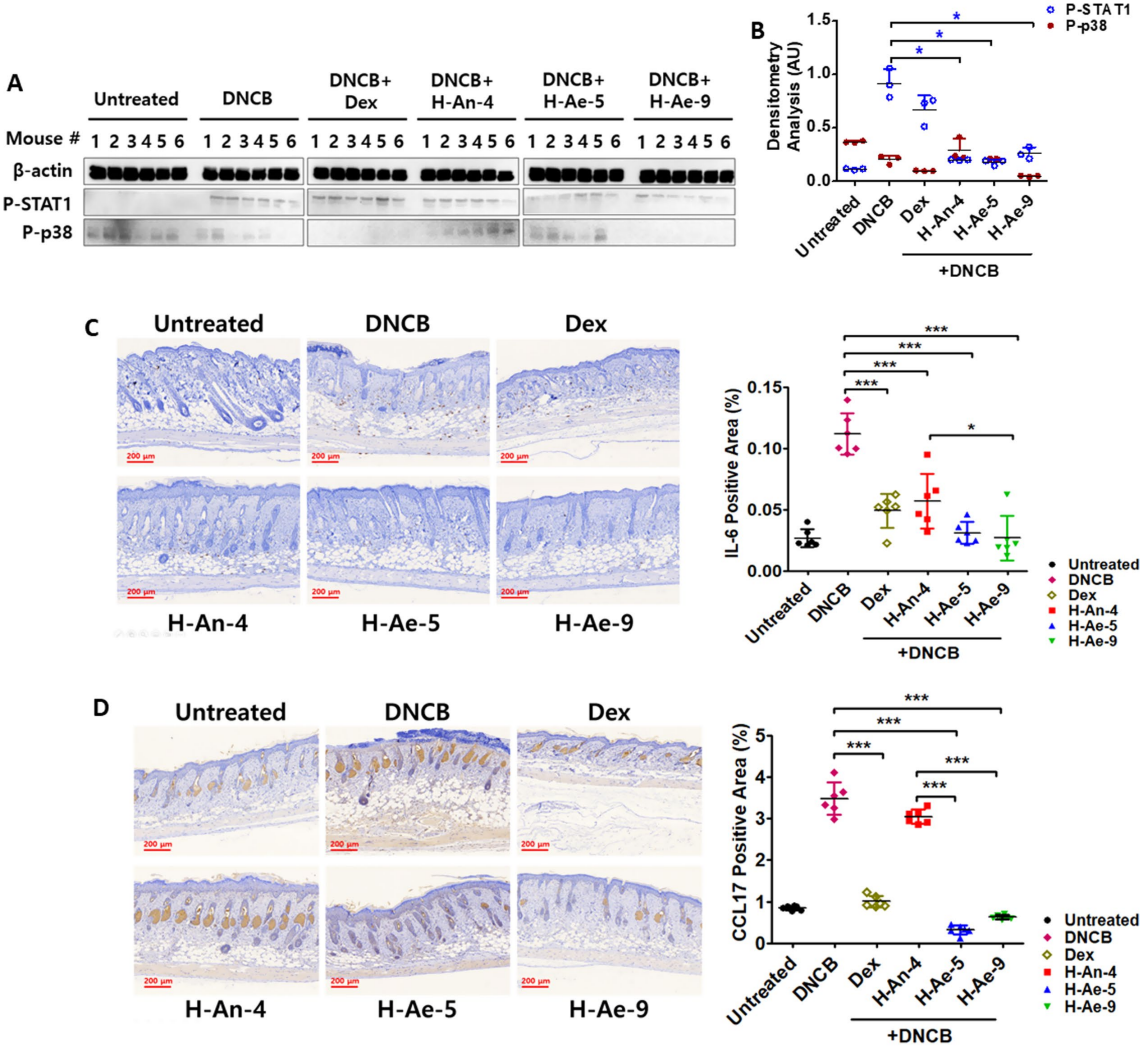
CS induces an anti-inflammatory response by inhibiting STAT1 and p38. Mouse dorsal skin samples were lysed and subjected to the WB analysis. The phosphorylation levels of STAT1 and p38 were measured **(A)**, and densitometric analysis was performed. P-STAT1 levels were significantly decreased (*p < 0.05) **(B)**. Immunofluorescence staining of mouse dorsal skin tissue was conducted to evaluate the expression of IL-6 **(C)** and CCL-17 **(D)**. The quantified positive area (%) of IL-6 (C) and CCL-17 (D) staining is shown in the right panels. Data are presented as mean ± SD. *p < 0.05, ***p < 0.001. Scale bars indicate 200 μm.

To investigate the molecular mechanisms underlying the anti-inflammatory effects of skin microbiota–derived CSs, the activation of key signaling molecules was examined in mouse skin tissues. As shown in Figure 6A, DNCB exposure markedly increased STAT1 phosphorylation compared to untreated controls. Among the treatment groups, STAT1 phosphorylation progressively decreased in the following order: H-An-4 CS > H-Ae-5 CS > H-Ae-9 CS > dexamethasone. In contrast, p38 phosphorylation was strongly suppressed in the H-Ae-9 CS- and dexamethasone-treated groups, indicating partial restoration of MAPK homeostasis. Quantitative densitometric analysis confirmed these trends (Figure 6B).

Immunohistochemical staining further demonstrated that DNCB-induced lesions exhibited elevated expression of IL-6 and CCL17, whereas treatment with bacterial CSs markedly reduced their levels (Figures 6C,D). Notably, treatment with H-Ae-5 CS and H-Ae-9 CS resulted in a pronounced decrease in IL-6 expression, while the inhibitory effects were less pronounced in the H-An-4 CS- and dexamethasone-treated groups. Similarly, CCL17 expression was most strongly reduced in the H-Ae-9 CS group, followed by the dexamethasone and H-Ae-5 CS groups, whereas H-An-4 CS showed no significant effect. The quantified positive area (%) of IL-6 (C) and CCL-17 (D) staining is shown in the right panels. DNCB markedly increased IL-6 and CCL-17 expression compared with the untreated group, whereas treatment with Dex or the indicated extracts (H-An-4, H-Ae-5, H-Ae-9) reduced the DNCB-induced elevation to varying degrees. Collectively, these results suggest that the anti-inflammatory effects of the bacterial CSs are associated with the modulation of p38 MAPK and STAT1 signaling pathways. The relatively modest changes observed *in vivo* may reflect the complexity of the skin microenvironment and differences in CS penetration or stability.

### Effects of bacterial culture supernatants on inflammation, neurogenic markers, and barrier-related gene expression in DNCB-induced atopic skin

**Figure 7:**
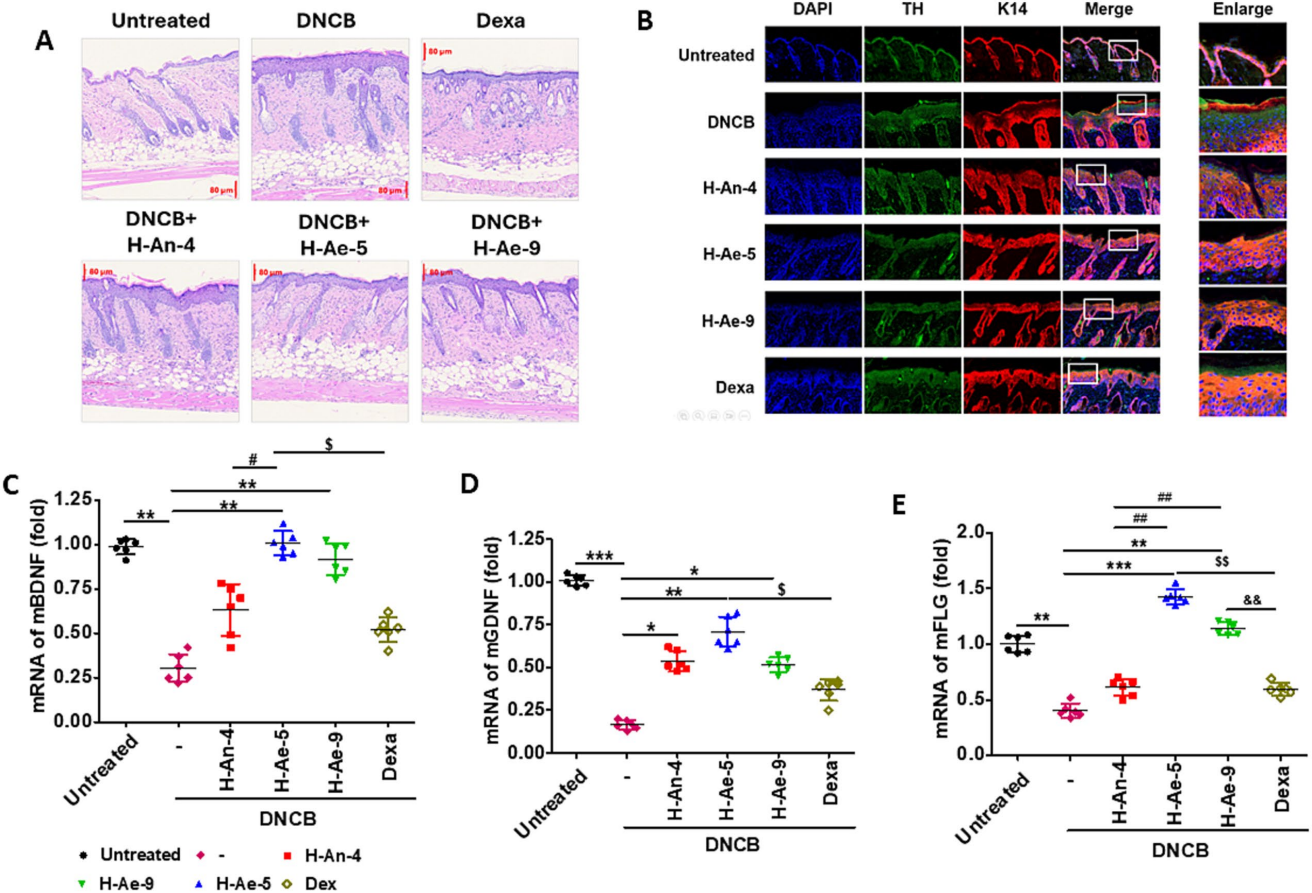
Histological, immunofluorescent, and gene expression analyses of DNCB-induced atopic dermatitis mouse skin treated with bacterial CSs. **(A)** H&E staining showing epidermal morphology and dermal cell infiltration in untreated, DNCB-treated, dexamethasone-treated, and bacterial CS–treated groups (H-An-4, H-Ae-5, H-Ae-9). Representative images are shown. Scale bars indicate 80 μm. **(B)** Immunofluorescence staining for tyrosine hydroxylase (TH) and keratin 14 (K14) in skin sections. DAPI was used for nuclear staining. Merged and enlarged images display co-localization patterns of TH and K14. **(C–E)** Quantitative RT-PCR analysis of BDNF **(C)**, GDNF **(D)**, and FLG **(E)** mRNA expression in mouse skin. Data are expressed as mean ± S.D. Statistical significance is indicated by the number of symbols (one symbol for *p* < 0.05, two for *p* < 0.01, and three for *p* < 0.001). The symbols denote comparisons as follows: * vs. DNCB; ^#^ Untreated vs. DNCB; $ H-Ae-5 vs. other groups; & H-Ae-9 vs. other groups.

Histological analysis using H&E staining revealed marked epidermal hyperplasia and dermal immune cell infiltration in DNCB-treated mice compared to untreated controls (Figure 7A). Treatment with dexamethasone (Dexa), used as a positive control, markedly attenuated these pathological changes, resulting in reduced epidermal thickness and inflammatory cell infiltration. Similarly, CSs derived from H-Ae-5 and H-Ae-9 substantially reduced inflammatory cell infiltration and restored near-normal epidermal architecture. In contrast, the H-An-4 CS-treated group showed only partial improvement, exhibiting a weaker anti-inflammatory effect compared to H-Ae-5 or H-Ae-9. Immunofluorescence analysis showed that DNCB markedly increased TH-positive sensory nerve fibers and induced epidermal hyperplasia (Figure 7B). Treatment with H-Ae-5 and H-Ae-9 substantially reduced TH levels and partially normalized epidermal structure, with H-Ae-9 exhibiting the most pronounced effect. In contrast, H-An-4 and dexamethasone did not effectively suppress TH overexpression; both groups retained elevated TH signals comparable to the DNCB group. These findings suggest that H-Ae-5 and H-Ae-9, but not H-An-4 or dexamethasone, attenuate DNCB-induced sensory nerve hyperactivation and neurogenic inflammation. The expression of the epithelial marker K14 remained largely unchanged across all groups.

RT-qPCR analysis using mouse skin tissues revealed that mRNA levels of brain-derived neurotrophic factor (BDNF) (Figure 7C), glial cell line-derived neurotrophic factor (GDNF) (Figure 7D), and filaggrin (FLG) (Figure 7E) were significantly reduced in DNCB-treated skin. Treatment with CSs from H-Ae-5 and H-Ae-9 substantially restored BDNF and GDNF expression and normalized FLG levels to near-control values. Among the bacterial CSs, H-Ae-5 CS exerted the strongest restorative effect, whereas H-An-4 CS produced only partial recovery. The positive control, dexamethasone, showed limited improvement, particularly for the neurotrophic factors. Collectively, these results demonstrate that culture supernatants from skin-resident bacteria, particularly H-Ae-5 and H-Ae-9, reduce inflammatory cell infiltration, suppress TH overexpression, and restore neurotrophic and barrier-related gene expression in DNCB-induced atopic skin. These findings suggest that bacterial metabolites contribute to neurocutaneous homeostasis by modulating immune, neural, and barrier pathways.

### Effects of bacterial culture supernatants on BDNF, GDNF, and NGF mRNA expression in differentiated SH-SY5Y cells via HaCaT- or HDF-conditioned media or direct treatment

**Figure 8:**
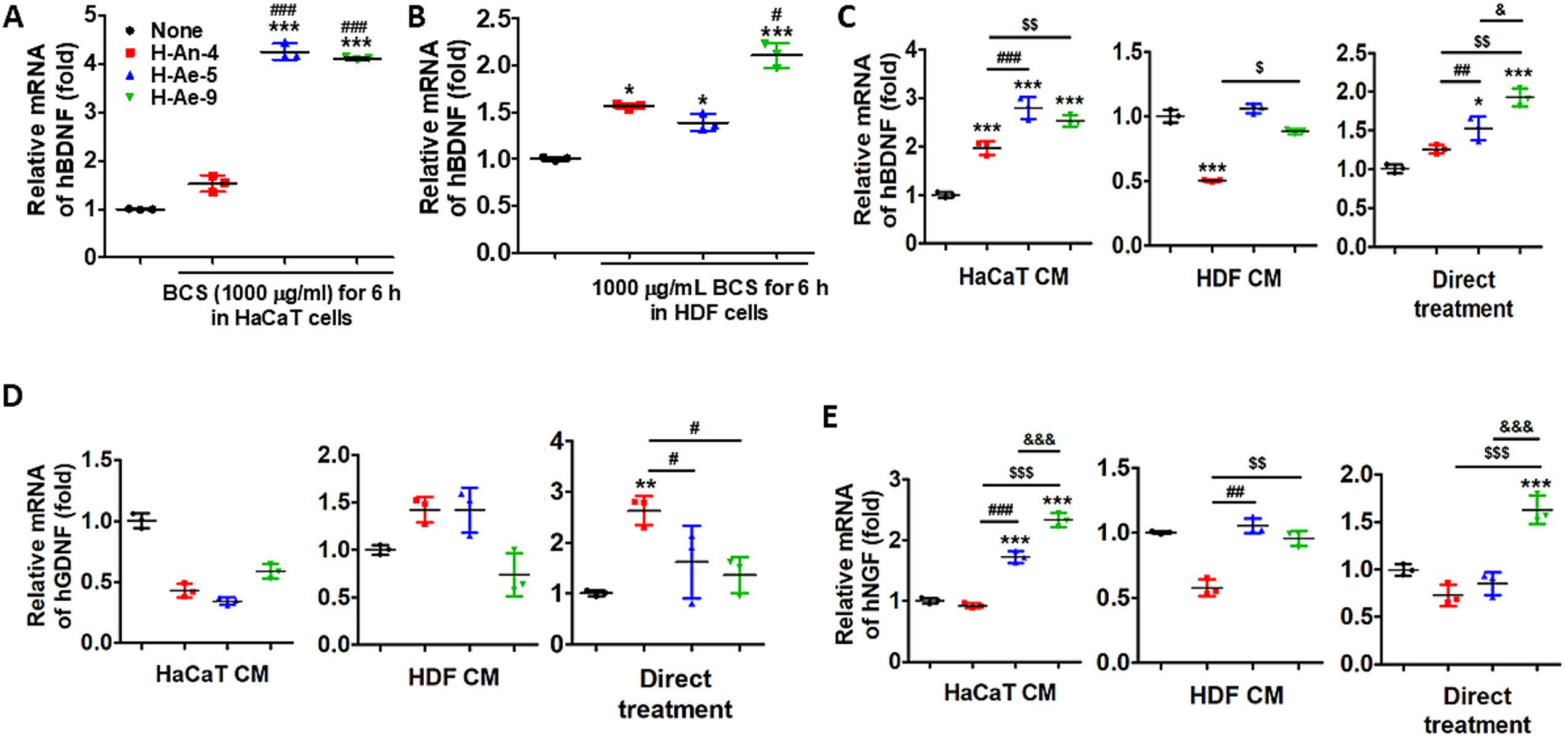
Bacterial CSs promote neurotrophic factor expression in both skin cells and differentiated SH-SY5Y cells. **(A)** HaCaT cells and **(B)** HDF cells were treated with each bacterial CS (1,000 μg/mL) for 6 h. Relative hBDNF mRNA levels were determined by qRT-PCR and normalized to the control (None). Differentiated SH-SY5Y cells were treated for 24 h with HaCaT CM, HDF CM, or direct bacterial CSs (H-An-4, H-Ae-5, H-Ae-9). Relative mRNA levels of **(C)** BDNF, **(D)** GDNF, and **(E)** NGF were quantified by qRT-PCR. Data are presented as mean ± S.D. Statistical significance is indicated by the number of symbols (one symbol for *p* < 0.05, two for *p* < 0.01, and three for *p* < 0.001). The symbols denote comparisons as follows: * vs. untreated control (None); ^#^ H-Ae-5 vs. H-An-4; $ H-Ae-9 vs. H-An-4; & H-Ae-9 vs. H-Ae-5.

Treatment with bacterial CSs significantly enhanced BDNF expression in both HaCaT and HDF cells (Figures 8A,B). In particular, CSs from H-Ae-5 and H-Ae-9 markedly upregulated BDNF mRNA levels (*p < 0.001), whereas H-An-4 CS did not induce significant changes in HaCaT cells. These results indicate that metabolites derived from H-Ae-5 and H-Ae-9 exert potent neurotrophic activity in both keratinocytes and dermal fibroblasts.

To evaluate the neurotrophic potential of bacterial CSs, differentiated SH-SY5Y neuronal cells were exposed for 24 h to HaCaT-conditioned medium (HaCaT CM), HDF-conditioned medium (HDF CM), or directly to the CSs. BDNF expression was significantly upregulated by HaCaT CM pretreated with CSs from H-Ae-5 and H-Ae-9, with a moderate increase observed following treatment with H-An-4 CS (Figure 8C). Treatment with HDF-conditioned medium (HDF CM) resulted in a significant suppression of BDNF expression by the H-An-4 CS, whereas CSs from H-Ae-5 and H-Ae-9 showed no noticeable effects. In SH-SY5Y cells, direct treatment with CSs from H-Ae-5 or H-Ae-9 enhanced BDNF expression, while H-An-4 CS did not induce any significant changes. GDNF expression remained largely unchanged under HaCaT- or HDF-conditioned medium treatments. However, a significant upregulation of GDNF was observed in SH-SY5Y cells directly treated with the H-An-4 CS (*p* < 0.01), whereas CSs from H-Ae-5 and H-Ae-9 did not induce significant changes. These results indicate that H-An-4-derived metabolites may exert a direct neurotrophic effect on neuronal cells (Figure 8D). NGF expression was significantly upregulated by CSs from H-Ae-5 and H-Ae-9 under all treatment conditions, with the most pronounced induction observed in HaCaT CM–treated cultures. In contrast, H-An-4 CS reduced NGF expression under the HDF CM condition, whereas direct treatment with H-Ae-9 led to a significant increase (*p* < 0.001) compared with the control (Figure 8E). Collectively, these results indicate that skin-resident bacterial culture supernatants enhance neurotrophic gene expression in differentiated neuronal cells both directly and indirectly through paracrine mechanisms mediated by epidermal and dermal cells. Among the tested strains, H-Ae-5 and H-Ae-9 exhibited the most potent neurotrophic activities.

## Discussion

Atopic dermatitis (AD) is a chronic inflammatory skin disorder affecting up to 20% of children and 10% of adults worldwide (Mocanu et al., 2021). Although corticosteroids remain central to symptom management, their prolonged use can cause adverse effects, including skin atrophy and dysregulated immunity in children (Mooney et al., 2015). These limitations underscore the need for safer alternatives that can control inflammation without compromising the skin barrier or immune homeostasis. The skin microbiome has emerged as a key regulator of cutaneous immunity, with AD patients exhibiting reduced microbial diversity and increased colonization by *Staphylococcus aureus* (Chng et al., 2016). Commensal microorganisms such as *Staphylococcus epidermidis* are known to suppress *S. aureus* and modulate inflammatory responses (Iwase et al., 2010; Alexander et al., 2020), suggesting that microbiome-derived factors may offer therapeutic benefits.

Accumulating evidence indicates that the skin microbiota plays an active role in shaping cutaneous immune responses in AD, rather than serving as a passive bystander. Dysbiosis in AD is characterized explicitly by reduced microbial diversity and functional alterations that directly influence host immunity (Chng et al., 2016). While Nakatsuji and Gallo (2019) reviewed these interactions, seminal work by Iwase et al. (2010) demonstrated that commensal bacteria, such as *Staphylococcus epidermidis*, can specifically suppress pathogenic *Staphylococcus aureus* colonization through the secretion of antimicrobial peptides like Esp. Furthermore, specific microbial communities have been shown to regulate keratinocyte-derived cytokines and chemokines, thereby influencing the type 2–skewed immune responses driving allergic skin inflammation (Koh et al., 2022; Alexander et al., 2020).

In addition to immune dysregulation, the disruption of epidermal barrier integrity is a central feature of AD, tightly linked to microbial imbalance. Reduced expression of barrier proteins, such as filaggrin, facilitates allergen penetration and microbial overgrowth, amplifying chronic inflammation. Koh et al. (2022) highlighted that alterations in skin microbiota composition are closely associated with impaired barrier function. Similarly, Mesjasz et al. (2023) emphasized that epidermal barrier defects and microbial dysbiosis act synergistically to perpetuate allergic inflammation. Importantly, our study aligns with the emerging view that microbial-derived metabolites can directly influence keratinocyte differentiation and barrier protein expression, providing a mechanistic link between microbial activity and epidermal homeostasis.

Beyond immune and barrier dysfunction, AD is increasingly recognized as a neuroimmune disorder where chronic pruritus and sensory nerve activation play pivotal roles. Cytokines associated with AD, including IL-31, directly modulate sensory neurons and glial cells, exacerbating itch and neurogenic inflammation (Matsuda et al., 2019). Furthermore, AD patients frequently exhibit neuropsychiatric comorbidities such as anxiety, depression, and sleep disturbances, underscoring the involvement of neuroimmune crosstalk (Yaghmaie et al., 2013). Recent functional MRI studies have further elucidated the neural correlates of pruritus and psychiatric symptoms in AD (Kim et al., 2022). Although these studies highlight the importance of neural regulation, the contribution of skin microbiota-derived signals to neurotrophic support—specifically via BDNF and GDNF—has remained poorly understood until now.

While live biotherapeutic approaches using commensal bacteria show promise, concerns regarding safety and stability persist, particularly with opportunistic species (Nakatsuji and Gallo, 2019). In this context, postbiotic strategies employing culture supernatants (CSs) offer a safer alternative. CS used in this study represent complex postbiotic preparations containing a mixture of secreted metabolites, peptides, and other bioactive molecules. Importantly, extracellular vesicles (EVs) are known to be present within bacterial culture supernatants and have recently been recognized as key mediators of microbiome–host communication. These vesicles carry diverse bioactive cargos, including proteins, lipids, and nucleic acids, and have been shown to modulate immune responses and barrier function in the context of atopic dermatitis. Therefore, it is plausible that the observed anti-inflammatory, barrier-restorative, and neuromodulatory effects of the CSs in this study may be partially mediated by EV-associated components, although this was not directly investigated. Given the multifactorial nature of host–microbe interactions, it is plausible that the observed biological effects are mediated by combined or synergistic actions of multiple components rather than a single dominant factor. Although the precise active constituents were not identified in the present study, our findings provide functional validation of the host-modulatory potential of these commensal-derived culture supernatants in both *in vitro* and *in vivo* settings. Our findings support this postbiotic paradigm by demonstrating that soluble metabolites from *B. paraconglomeratum* and *B. casei* can recapitulate immunomodulatory, barrier-restorative, and neurotrophic effects without the risks associated with live bacterial administration. Future studies employing bioactivity-guided fractionation, metabolomic profiling, and vesicle isolation approaches will be required to define the relative contribution of soluble metabolites and EV-associated factors to the observed biological effects.

**Figure 9:**
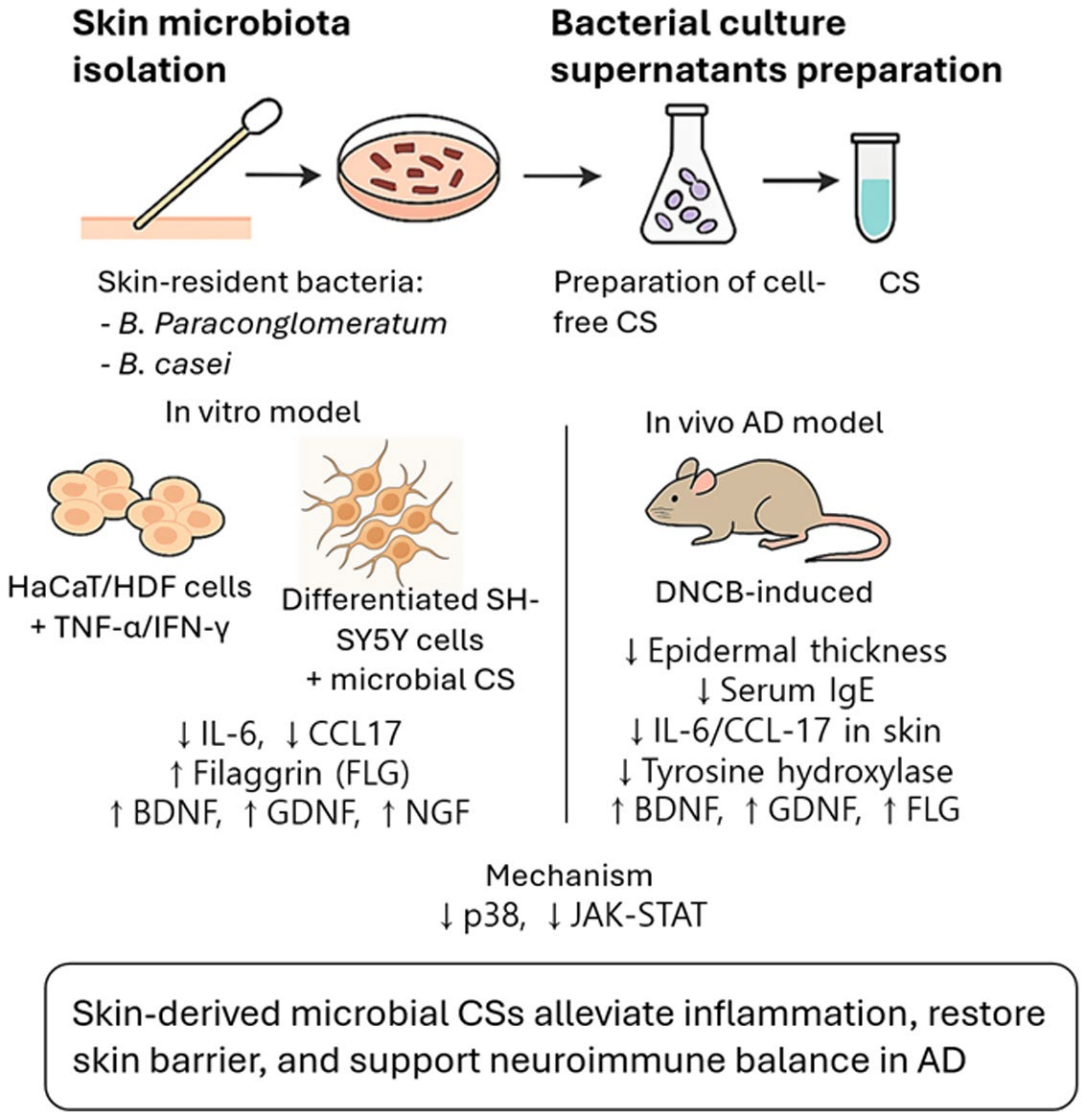
Proposed working model of skin microbiota-derived culture supernatants in alleviating atopic dermatitis. CSs derived from human skin-resident *B. paraconglomeratum* and *B. casei* exert therapeutic effects across the skin-neuroimmune axis. In the *in vitro* models, the microbial CSs inhibit pro-inflammatory cytokines (IL-6, CCL17) via suppression of p38 MAPK and JAK–STAT pathways, while upregulating barrier proteins (FLG) and neurotrophic factors (BDNF, GDNF, NGF). In the *in vivo* DNCB-induced AD model, topical application of these CSs restores epidermal barrier integrity, suppresses systemic IgE and local inflammation, and modulates neurogenic markers (decreasing TH and increasing BDNF/GDNF/FLG). Collectively, this cell-free postbiotic strategy effectively restores neuroimmune balance and alleviates AD pathogenesis.

In this study, we demonstrate that soluble metabolites derived from skin-resident Actinobacteria can modulate cutaneous inflammation and neurocutaneous signaling in AD models, even in the absence of live bacteria. This represents a significant conceptual shift from live microbial therapeutics toward postbiotic approaches that harness microbial bioactivity while minimizing risks associated with colonization or dysbiosis. Conditioned supernatants (CSs) from H-Ae-5 (*B. paraconglomeratum*) and H-Ae-9 (*B. casei*) suppressed cytokine expression in TNF-α/IFN-γ–stimulated HaCaT cells and alleviated DNCB-induced AD-like symptoms in vivo. These effects were accompanied by reduced activation of p38 MAPK and STAT1, suggesting a potential involvement of these pathways in mediating the anti-inflammatory effects (Pastore et al., 2010). While p38 MAPK and STAT1 signaling pathways were modulated by CS treatment, the present data do not establish a direct causal relationship, and further studies will be required to define the precise molecular mechanisms underlying the observed effects. Importantly, the CSs also restored FLG, BDNF, and GDNF expression while reducing tyrosine hydroxylase (TH) levels in AD skin, suggesting that postbiotic metabolites influence not only immune signaling but also epithelial–neuronal–immune interactions that contribute to pruritus and tissue dysfunction (Ulmann et al., 2010) (Figure 9).

Although *B. paraconglomeratum* and *B. casei* have previously been described as opportunistic species (Bonavila Juana et al., 2017; Tamai et al., 2018), our findings reveal that their metabolites exert beneficial immunomodulatory effects under non-pathogenic conditions. Unlike well-studied skin commensals such as *S. epidermidis*, the therapeutic potential of cutaneous *Actinobacteria* has remained largely unexplored. Interestingly, our study showed that CSs from *Actinobacteria* strains (H-Ae-5, H-Ae-9) exhibited superior anti-inflammatory effects compared to the *Cutibacterium* strain (H-An-4). This differential efficacy may stem from distinct metabolic profiles; for instance, *Actinobacteria* are known to produce unique secondary metabolites, including specific antibiotics and immunomodulatory peptides, which may target host receptors differently than those from *Firmicutes* (Christensen and Brüggemann, 2014). Our study explicitly focused on uncovering the functional roles of these novel candidates to expand the repertoire of beneficial skin microbiota. This supports the emerging view that the skin microbiome includes conditionally beneficial taxa whose functional outputs—rather than their mere presence—shape immune homeostasis. Because CSs lack viable bacteria, they circumvent the safety concerns traditionally associated with opportunistic or less-characterized strains, providing a practical means to leverage microbial functionality without the risks associated with live-cell application.

Notably, the extent of cytokine suppression varied between *in vitro* and *in vivo* systems. These discrepancies may result from differences in CS stability, penetration through the stratum corneum, or the complexity of cell–cell interactions within intact skin. Regarding the experimental models, we utilized TNF-α/IFN-γ stimulation in vitro to mimic the broad inflammatory milieu of the chronic phase of AD, where Th1 cytokines act as key amplifiers of barrier disruption (Nomura et al., 2003). Similarly, while the DNCB-induced in vivo model primarily reflects acute contact dermatitis rather than the chronic relapsing nature of human AD, it serves as a robust screening platform for evaluating the anti-inflammatory potential of novel postbiotic candidates. Furthermore, we observed a dissociation between the significant reduction in serum IgE levels and the persistence of splenomegaly in the CS-treated groups. Splenomegaly in the DNCB model typically arises from the systemic expansion and recruitment of immune cells. While our bacterial CSs effectively suppressed the functional differentiation of B cells—evidenced by the sharp decline in serum IgE—the anatomical regression of splenic hypertrophy may require a prolonged recovery phase exceeding the experimental duration. This suggests that the therapeutic action of the CSs primarily targets inflammatory signaling cascades and functional immune polarization rather than inducing immediate clearance of the hyperplastic immune cell population. Nevertheless, both models consistently demonstrated downregulation of inflammatory signaling, highlighting the robustness of the observed mechanism. While the DNCB-induced model primarily reflects acute contact dermatitis rather than the chronic nature of human AD, it serves as a robust screening platform for evaluating the anti-inflammatory potential of novel postbiotic candidates. Future studies should incorporate metabolomic profiling to identify the active molecules within CSs, as well as controlled human skin models to validate their safety, diffusion dynamics, and efficacy in clinically relevant settings.

Finally, we acknowledge several limitations in this study that outline important directions for future research. First, although we demonstrated the efficacy of crude CSs, the specific active components remain to be identified. Given that postbiotic activity often arises from the synergistic effects of complex metabolites, this study serves as a proof-of-concept for the therapeutic potential of the overall secretome. Future metabolomic profiling will be essential to fractionate and identify specific bioactive molecules. Second, although we observed modulation of neurotrophic factors (BDNF, GDNF) and signaling pathways (p38, STAT1), the upstream receptors (e.g., TLRs or GPCRs) responsible for initiating these responses were not investigated. In addition, functional behavioral assessments, such as pruritus-related scratching behavior, were not included. Future studies using pathway-specific inhibitors, receptor blocking approaches and behavioral analyses will be required to clarify the underlying mechanisms. While the restoration of neurotrophic factors such as BDNF and GDNF suggests a potential role in modulating neuroimmune interactions, the present study does not establish whether these changes act as primary drivers of therapeutic effects or occur as secondary consequences of reduced inflammation. It is plausible that the observed neurotrophic alterations reflect a bidirectional interaction between immune regulation and neuronal signaling. Further studies will be required to delineate the causal relationship between neurotrophic modulation and disease improvement. Third, further validation in chronic AD models and human skin explants is needed to better assess the long-term efficacy and translational potential of these postbiotics preparations.

Overall, this study positions skin microbiota-derived postbiotics as a promising therapeutic platform for AD. By modulating inflammatory, neurotrophic, and barrier-related pathways, postbiotic metabolites offer a safer, more stable, and more regulatory-friendly alternative to live biotherapeutics (Salminen et al., 2021). These findings expand the current understanding of microbiome–host interactions and provide a foundation for developing next-generation, microbiome-balanced interventions for chronic inflammatory skin diseases.

## Data Availability

The original contributions presented in the study are included in the article/Supplementary material, further inquiries can be directed to the corresponding authors.
