# Supplementary material for: Culture supernatants from human-derived commensal bacteria alleviate DNCB-induced atopic dermatitis through modulation of inflammatory and barrier-associated pathways
Source: Front Microbiol. 2026 May 1;17:1813592. doi: 10.3389/fmicb.2026.1813592 (PMC13176294; doi:10.3389/fmicb.2026.1813592)
Supplement: Supplementary file 1 [file Data_Sheet1.docx]

**Supplementary Fig. 1. Screening of anti-inflammatory activity of skin-derived bacterial isolates.**


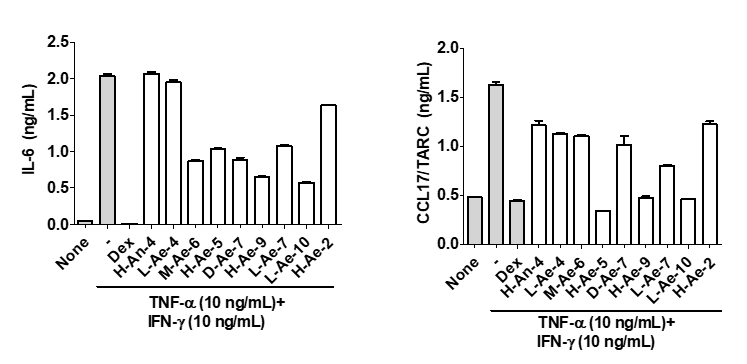


Keratinocytes were treated with culture supernatants (CSs) derived from nine skin-resident bacterial isolates, followed by stimulation with TNF-α (10 ng/mL) and IFN-γ (10 ng/mL). The levels of pro-inflammatory cytokines IL-6 and CCL17 were measured to evaluate anti-inflammatory activity. Among the tested strains, H-Ae-5 (Brachybacterium paraconglomeratum) and H-Ae-9 (Brevibacterium casei) exhibited the most consistent and pronounced inhibitory effects and were therefore selected for further investigation.

**Supplementary Fig. 2. Summary of physiological parameters during the experimental period.**

**(A) Food consumption:**

Weekly food intake remained stable across all groups, with no noticeable differences between control, DNCB-treated, dexamethasone-treated, or bacterial administration groups (*C. acnes*, *B. paraconglomeratum*, *B. casei*).

**(B) Body weight:**

Body weight gradually increased over the 4-week period in all groups. No treatment, including DNCB challenge or probiotic administration, caused significant weight loss or adverse effects.

**(C) Skin hydration level:**

DNCB treatment markedly reduced skin hydration from Week 1 onward, confirming successful induction of atopic-like dryness. Treatment with *B. paraconglomeratum* and *B. casei* partially restored hydration levels, whereas *C. acnes* showed modest effects. Dexamethasone did not normalize hydration levels despite reducing inflammation.
